# Genetic, environmental, and behavioral correlates of lifetime suicide attempt: Analysis of additive and interactive effects in two cohorts of US Army soldiers

**DOI:** 10.1038/s41386-023-01596-2

**Published:** 2023-05-19

**Authors:** Laura Campbell-Sills, Xiaoying Sun, Santiago Papini, Karmel W. Choi, Feng He, Ronald C. Kessler, Robert J. Ursano, Sonia Jain, Murray B. Stein

**Affiliations:** 1 Department of Psychiatry, University of California San Diego, La Jolla, CA USA; 2 Herbert Wertheim School of Public Health and Human Longevity Science, University of California San Diego, La Jolla, CA USA; 3 Division of Research, Kaiser Permanente Northern California, Oakland, CA USA; 4 Center for Precision Psychiatry, Department of Psychiatry, Massachusetts General Hospital, Boston, MA USA; 5 Psychiatric and Neurodevelopmental Genetics Unit, Center for Genomic Medicine, Massachusetts General Hospital, Boston, MA USA; 6 Stanley Center for Psychiatric Research, Broad Institute, Boston, MA USA; 7 Department of Health Care Policy, Harvard Medical School, Boston, MA USA; 8 Center for the Study of Traumatic Stress, Department of Psychiatry, Uniformed Services University of the Health Sciences, Bethesda, MD USA; 9 VA San Diego Healthcare System, San Diego, CA USA

**Keywords:** Risk factors, Behavioural genetics

## Abstract

Recently developed measures of genetic liability to suicide attempt may convey unique information regarding an individual’s risk of suicidal behavior. We calculated a polygenic risk score for suicide attempt (SA-PRS) for soldiers of European ancestry who participated in the Army STARRS New Soldier Study (NSS; *n* = 6573) or Pre/Post Deployment Study (PPDS; *n* = 4900). Multivariable logistic regression models were fit within each sample to estimate the association of SA-PRS with lifetime suicide attempt (LSA), and to examine whether SA-PRS displayed additive or interactive effects with environmental and behavioral risk/protective factors (lifetime trauma burden, childhood maltreatment, negative urgency impulsivity, social network size, perceived mattering, and dispositional optimism). Age, sex, and within-ancestry variation were included as covariates. Observed prevalence of LSA was 6.3% and 4.2% in the NSS and PPDS samples, respectively. In the NSS model, SA-PRS and environmental/behavioral factors displayed strictly additive effects on odds of LSA. Results indicated an estimated 21% increase in odds of LSA per 1 SD increase in SA-PRS [adjusted odds ratio (AOR; 95% CI) = 1.21 (1.09–1.35)]. In PPDS, the effect of SA-PRS varied by reports of optimism [AOR = 0.85 (0.74–0.98) for SA-PRS x optimism effect]. Individuals reporting low and average optimism had 37% and 16% increased odds of LSA per 1 SD increase in SA-PRS, respectively, whereas SA-PRS was not associated with LSA in those reporting high optimism. Overall, results suggested the SA-PRS had predictive value over and above several environmental and behavioral risk factors for LSA. Moreover, elevated SA-PRS may be more concerning in the presence of environmental and behavioral risk factors (e.g., high trauma burden; low optimism). Given the relatively small effect magnitudes, the cost and incremental benefits of utilizing SA-PRS for risk targeting must also be considered in future work.

## Introduction

Suicidal behavior is a major public health problem in the US, with approximately 12.2 million adults seriously contemplating suicide, 1.2 million attempting suicide, and 45,000 dying by suicide in 2020 alone [1]. These events cause enormous personal suffering and carry high societal costs [2]; and, despite decades of research, major advancements in the prediction and prevention of suicidal behavior remain elusive [3]. Further insights into the etiology of suicidal behavior and innovative tools for risk stratification are needed to enhance these capabilities. The etiology of suicidal behavior is complex, with risk and protective factors exhibiting distinct relationships with the outcomes of suicidal ideation, suicide attempt, and suicide death [4]. An implication of this is that it is important for scientific inquiry to focus on vulnerability to specific forms of suicidal behavior, as well as mechanisms involved in the progression from contemplating suicide to formulating a plan and/or making an attempt [5, 6]. The current study targets the former objective by examining the association of a specific polygenic risk score for suicide attempt (SA-PRS) with the phenotype of lifetime suicide attempt (LSA).

 Wide-ranging evidence implicates genetic variation in the etiology of suicide attempt [4, 7, 8]. Genomewide association studies (GWAS) indicate suicide attempt is a significantly heritable outcome with a polygenic basis, and that the genetic architecture of suicide attempt and psychiatric disorders partly overlaps [9–15]. The converse observation–that some of the genetic risk for suicide attempt is independent of genetic vulnerability to psychiatric disorders–implies that specific measures of genetic risk for suicide attempt have the potential to contribute valuable information for suicide risk classification. Emerging evidence supports this supposition, with initial studies showing that SA-PRS differentiate suicide attempt cases versus controls among adults with mood disorders and schizophrenia [12]. SA-PRS have also been shown to predict suicide attempt among children and adolescents, independent of effects of other risk factors such as other psychiatric polygenic risk scores (PRS), family history of suicidal behavior, and measures of temperament and psychopathology [16, 17].

Also relevant to the current study is recent research using a broadly defined suicidality PRS, which quantifies genetic risk for passive suicidal ideation, contemplating self-harm or suicide, non-suicidal self-injury, or attempted suicide [13]. Investigations using this suicidality PRS have found moderating effects of environmental and behavioral factors on the associations between that PRS and suicide-related outcomes. One study demonstrated that the increased risk of suicide attempt associated with higher suicidality PRS was magnified in the presence of high trauma exposure [18]. Another found that dispositional optimism and social support each buffered the effects of higher suicidality PRS on risk of suicidal ideation outcomes [19].

To build on these informative initial studies, we tested the hypothesis that an SA-PRS would exhibit significant associations with LSA in two cohorts of US Army soldiers. We further examined whether the associations of SA-PRS with LSA were moderated by environmental and behavioral factors. Selection of the environmental and behavioral variables was based on the aforementioned empirical results [18, 19] and on theories of suicidal behavior that highlight individual differences in personality, stress exposure (particularly early life adversity), social cohesion, and cognitive style [4, 6, 20]. Specifically, we evaluated whether higher levels of lifetime trauma, childhood maltreatment, and impulsivity potentiated the effect of SA-PRS on risk of LSA; and whether more robust social networks, dispositional optimism, and perceptions of mattering to other people buffered the effect of SA-PRS on risk of LSA.

## Methods and Materials

### Overview and participants

The data analyzed in this study come from two components of the Army Study to Assess Risk and Resilience in Servicemembers [Army STARRS; [21, 22]]. The New Soldier Study (NSS) was conducted from April 2011 to November 2012 at three US Army installations. Consenting soldiers self-administered the computerized NSS survey before Basic Combat Training. The Pre/Post Deployment Study (PPDS) was a multi-wave panel survey of three US Army Brigade Combat Teams that deployed to Afghanistan in 2012. The PPDS baseline (T0) survey was administered 1–2 months before deployment, and follow-up survey data were collected approximately 1 month (T1), 3 months (T2), and 9 months (T3) after return from deployment. The NSS and PPDS T0 surveys assessed socio-demographic characteristics, lifetime and past-30-day mental disorders and suicidality, and risk and resilience factors. The PPDS T1, T2, and T3 surveys focused on experiences and symptoms that had occurred during and since return from the index deployment. Participants provided written informed consent to participate in each survey, to link their survey data and Army/Department of Defense (DoD) administrative records, and to provide blood samples for Army STARRS biomarkers studies. Study procedures were approved by the Human Subjects Committees at the collaborating institutions (including the Uniformed Services University of the Health Sciences for the Henry M. Jackson Foundation; the Institute for Social Research at the University of Michigan, Ann Arbor; Harvard Medical School; and University of California San Diego).

The NSS survey was completed by 39,784 soldiers. A total of 33,088 (83.2%) gave blood samples; however, due to resource constraints only 10,529 were genotyped. All soldiers reporting LSA or lifetime PTSD on the NSS survey were genotyped, along with a set of control respondents matched on key characteristics [23]. The PPDS T0 survey was completed by 9488 soldiers, of whom 7625 (80.4%) gave blood samples and were genotyped. Analysis samples for the current study were constrained to soldiers of genetically determined European ancestry, given limited availability of reference GWAS data in other populations. This final constraint yielded *n* = 6573 for NSS analyses and *n* = 4900 for PPDS analyses. We had access to individual-level genetic data for all participants and used this to verify that there was no overlap between the two analysis samples.

### Measures

#### Lifetime suicide attempt

Two sources were used to determine LSA status: survey data and Army/DoD administrative records. In the NSS and PPDS surveys, suicidal thoughts and behaviors were assessed using an expanded self-report version of the Columbia-Suicide Severity Rating Scale [C-SSRS; [24]]. The item assessing LSA inquired whether the respondent had ever made “a suicide attempt (i.e., purposefully hurt yourself with at least some intention to die)*”*. Additional C-SSRS data were available for NSS and PPDS respondents who participated in wave 1 of the STARRS Longitudinal Study [STARRS-LS1; conducted September 2016 to April 2018; [25]; these data were also considered in determining LSA case status. Finally, information regarding LSA was available from Army/DoD records (covering the years 2005–16) that were compiled for the Army STARRS Historical and Administrative Data Study [22].

In the current study, LSA was considered present if either of the following two conditions were met: (1) the respondent gave an affirmative response to the C-SSRS suicide attempt item in any Army STARRS survey, regardless of the timeframe referenced (e.g., at any time in their life in the NSS and PPDS T0 surveys; since their last survey in the PPDS T2/T3 and STARRS-LS1 surveys); or (2) any of their Army/DoD records indicated that they had made a suicide attempt. There were 24 respondents missing LSA data in NSS and 2 respondents missing LSA data in PPDS; their status was imputed as “No” given that it is far more likely for a given individual to have never *vs* ever attempted suicide (i.e., low base rate of LSA).

#### Polygenic risk score for suicide attempt (SA-PRS)

Army STARRS methods for DNA collection, genotyping, quality control, and ancestry assignment are described in detail elsewhere [26]. For the current study, summary statistics from a published GWAS of suicide attempt [[12]; *N* = 538,436 after excluding PPDS and NSS cohorts] and a European ancestry reference panel were used to estimate SNP effect sizes with PRS-CS-auto [27], and PLINK 2.0 [28] was used to estimate the SA-PRS (standardized within each sample).

#### Lifetime trauma burden

The NSS and PPDS T0 surveys assessed lifetime exposure to potentially traumatic events (PTEs) including physical assault, sexual assault or rape, serious assault that happened to a loved one, other life-threatening experience that happened to a loved one, traumatic death of a loved one (due to murder, combat, or accident), witnessing someone being seriously injured or killed, discovering or handling a dead body, life-threatening illness or injury, being in a natural disaster that put one at risk of death or serious injury, other life-threatening experience, and being bullied as a child or adolescent. Responses to PTE items were coded as present (“1 time” to “10 or more times”) or absent (“0 times”) and summed to create a score representing lifetime trauma burden (theoretical range = 0–13, higher scores indicate more types of PTE exposure). Two items assessing suicide and attempted suicide of “close friends or relatives” were excluded from the trauma burden score due to potential overlap with the SA-PRS (i.e., suicidal behavior among relatives could indicate genetic risk for suicide attempt).

#### Childhood maltreatment

The assessment of childhood maltreatment in Army STARRS surveys is described in detail elsewhere [29]. Here we used a global maltreatment scale, which captures exposure to sexual abuse, physical abuse, emotional abuse, physical neglect, and emotional neglect through age 18 (theoretical range = 1–5, higher scores indicate more extensive or frequent maltreatment).

#### Social network size

The NSS and PPDS T0 surveys asked, “How many people do you have in your personal life of the following sorts?… (1) People you do things with, like watch TV together, go out for a drink or movie together, or play cards; (2) people who you feel really close to, (3) people who really care for you and would be there if you needed them, and (4) family or friends who need you and rely on you for help when they need it.” The 4 items were rated on a 10-point scale with categories ranging from “0” to “31 or more” people. Following a prior study that linked scores on this measure to future suicidal behavior [30], item ratings were recoded 0–9 and summed to provide an overall measure of social network size (theoretical range = 0–36, with higher scores indicating larger social networks; NSS α = 0.81, PPDS α = 0.85).

#### Personality variables

The NSS and PPDS T0 surveys assessed personality traits using brief scales comprised of items adapted from validated self-report inventories [31]. The scales of interest for this study were negative urgency impulsivity (2 items; e.g., “When I am upset I often act without thinking”; NSS α = 0.48, PPDS α = 0.60), perceived mattering (2 items; e.g., “I bring a lot of happiness to the people in my life”; NSS α = 0.86, PPDS α = 0.90) and dispositional optimism (2 items; e.g., “I usually look on the bright side of things”; NSS α = 0.44, PPDS α = 0.63).

### Data analysis

Statistical analyses were conducted in R version 3.6.1 [32]. Pearson’s correlation coefficients were calculated to evaluate associations among the predictors of interest (SA-PRS, lifetime trauma burden, childhood maltreatment, negative urgency impulsivity, social network size, perceived mattering, and dispositional optimism). In addition to signaling any possible multicollinearity concerns, these correlations allowed us to rule out potential PRS x environment associations that could complicate interpretation of the study findings. Univariate associations between the predictors of interest and the outcome were subsequently assessed using t-tests. Finally, a multivariable logistic regression model of LSA was fit within each sample to evaluate additive and interactive effects of SA-PRS and the environmental and behavioral risk/protective factors. We first specified a preliminary model that included main effects of predictors of interest (and covariates), and interactions between SA-PRS and the other predictors of interest. We then eliminated any non-significant interaction terms yielding a simplified final model of LSA within each sample. Measures of genetic, environmental, and behavioral risk/protective factors were standardized prior to performing logistic regression, to facilitate interpretation of the adjusted odds ratios (AORs). All models adjusted for age and sex (self-reported on the NSS or PPDS T0 survey), within-ancestry variation using 10 principal components [33] and tranche (for NSS models only, as the NSS samples had been genotyped in two tranches). Two-tailed *p*-values < 0.05 were considered statistically significant.

## Results

The socio-demographic characteristics of the study samples are displayed in Table 1. Table 2 shows descriptive statistics and correlations among the hypothesized risk and protective factors, stratified by sample. SA-PRS was not significantly correlated with any environmental or behavioral factor in either sample (*r* = −0.04 to 0.05). Most correlations among the environmental and behavioral factors were negligible or small, although a few medium-sized correlations were observed among the protective factors (*r* = 0.30–0.42).

**Table 1:** Socio-demographic characteristics of the New Soldier Study (*n* = 6573) and Pre/Post Deployment Study (*n* = 4900) samples.

	New Soldier Study		Pre/Post Deployment Study	
	No.	%	No.	%
Age, years (mean, SD)	20.8	3.3	25.9	5.9
Male	5600	85.2	4676	95.7
Female	973	14.8	208	4.3
GED/equivalent	690	10.5	350	7.2
High school diploma	5473	83.3	3557	73.2
College degree	410	6.2	955	19.6
Never married	5825	88.6	1770	36.6
Married	742	11.3	2614	54.0
Divorced/Separated/Widowed	6	0.1	456	9.4
Regular Army	3712	57.8	4900	100
Army Reserve	767	11.9	0	0
Army National Guard	1946	30.3	0	0

Samples were restricted to soldiers of genetically determined European ancestry; thus, race and ethnicity characteristics are not reported. Due to small amounts of missing data, some socio-demographic categories may not sum to the total sample size.

**Table 2:** Means and correlations of risk/protective factors in New Soldier Study (*n* = 6573) and Pre/Post Deployment Study (*n* = 4900) samples.

	Range	Mean (SD)	SA-PRS	Maltreat	Trauma burden	Negative urgency	Social network	Optimism	Mattering
**NSS sample**									
SA-PRS	std	0.00 (1.00)	1.00						
Maltreatment	1–5	1.55 (0.65)	0.03	1.00					
Trauma burden	0–13	3.28 (2.78)	0.04	0.23	1.00				
Negative urgency	0–8	2.99 (2.04)	0.01	0.10	0.12	1.00			
Social network	0–36	18.32 (7.26)	−0.04	−0.22	0.06	−0.01	1.00		
Optimism	0–8	4.87 (1.94)	0.00	−0.06	0.02	−0.02	0.15	1.00	
Mattering	0–8	5.83 (1.88)	0.01	−0.19	−0.01	−0.03	0.26	0.34	1.00
**PPDS sample**									
SA-PRS	std	0.00 (1.00)	1.00						
Maltreatment	1–5	1.31 (0.52)	0.05	1.00					
Trauma burden	0–13	3.17 (2.82)	0.02	0.27	1.00				
Negative urgency	0–8	2.01 (1.86)	0.04	0.16	0.16	1.00			
Social network	0–36	16.53 (7.46)	−0.04	−0.24	−0.02	−0.09	1.00		
Optimism	0–8	4.86 (2.13)	−0.01	−0.16	−0.02	−0.03	0.25	1.00	
Mattering	0–8	5.73 (1.99)	0.00	−0.26	−0.06	−0.09	0.30	0.42	1.00

*NSS* New Soldier Study, *PPDS* Pre/Post Deployment Study, *SA-PRS* Polygenic risk score for suicide attempt, *std* Standardized.

The observed prevalence of LSA within NSS and PPDS samples was 6.3% and 4.2%, respectively. Univariate tests of association indicated that all hypothesized risk and protective factors were significantly associated with LSA in both samples (Table 3). Compared to soldiers without LSA, those with a history of LSA had higher SA-PRS, lifetime trauma burden, childhood maltreatment, and negative urgency impulsivity scores, and lower scores on the measures of social network size, perceived mattering, and dispositional optimism.

**Table 3:** Univariate associations of hypothesized risk and protective factors with lifetime suicide attempt in the two samples.

	NSS sample (*n* = 6573)			PPDS sample (*n* = 4900)		
	LSA = No (*n* = 6156)	LSA = Yes (*n* = 417)	*p*-value for difference test	LSA = No (*n* = 4696)	LSA = Yes (*n* = 204)	*p*-value for difference test
SA-PRS	−0.012 (1.00)	0.18 (0.93)	< 0.001	−0.009 (1.00)	0.20 (0.97)	0.003
Maltreatment	1.52 (0.62)	1.93 (0.86)	< 0.001	1.30 (0.50)	1.64 (0.77)	< 0.001
Trauma burden	3.21 (2.74)	4.30 (3.13)	< 0.001	3.11 (2.79)	4.53 (3.23)	< 0.001
Negative urgency	2.93 (2.02)	3.88 (2.15)	< 0.001	1.97 (1.83)	3.03 (2.21)	< 0.001
Social network	18.47 (7.20)	16.06 (7.82)	< 0.001	16.58 (7.43)	15.23 (8.01)	0.020
Optimism	4.90 (1.93)	4.34 (2.04)	< 0.001	4.87 (2.12)	4.50 (2.32)	0.026
Mattering	5.88 (1.86)	5.19 (2.16)	< 0.001	5.75 (1.98)	5.24 (2.13)	< 0.001

Values are mean (SD). *NSS* New Soldier Study, *PPDS* Pre/Post Deployment Study, *LSA* Lifetime suicide attempt, *SA-PRS* Polygenic risk score for suicide attempt.

### Multivariable logistic regression model of LSA in the NSS sample

The multivariable model of LSA within the NSS sample is shown in Table 4 (panel A). None of the interaction terms approached significance in the preliminary model (AORs = 0.96–1.01, *p*s > 0.38); thus, the final model estimated only main effects of SA-PRS and environmental and behavioral risk/protective factors. Results indicated that SA-PRS was associated with LSA, with an estimated 21% increase in odds of LSA per 1 SD increase in SA-PRS [AOR (95% CI) = 1.21 (1.09–1.35), X^2^ = 12.49, *p* < 0.001]. Higher lifetime trauma burden [AOR = 1.25 (1.13–1.38), X^2^ = 18.70, *p* < 0.001), childhood maltreatment [AOR = 1.36 (1.25–1.49), X^2^ = 47.15, *p* < 0.001], and negative urgency impulsivity [AOR = 1.38 (1.24–1.53), X^2^ = 37.46, *p* < 0.001] were also associated with significantly increased odds of LSA. Conversely, greater social network size [AOR = 0.84 (0.76–0.94), X^2^ = 9.19, *p* = 0.002], perceived mattering [AOR = 0.84 (0.75–0.93), X^2^ = 10.79, *p* = 0.001], and dispositional optimism [AOR = 0.85 (0.76–0.95), X^2^ = 8.35, *p* = 0.004] were associated with significantly reduced odds of LSA.

**Table 4:** Multivariable models of lifetime suicide attempt in (A) the New Soldier Study sample and (B) the Pre/Post Deployment Study sample.

	A.NSS sample (*n* = 6573)	B.PPDS sample (*n* = 4900)
Age (years)	0.94 (0.90–0.97)**	0.97 (0.94–0.99)*
Female sex (reference: male)	1.18 (0.89–1.55)	1.72 (0.98–3.04)
Lifetime trauma burden	1.25 (1.13–1.38)***	1.40 (1.22–1.61)***
Childhood maltreatment	1.36 (1.25–1.49)***	1.25 (1.13–1.40)***
Negative urgency impulsivity	1.38 (1.24–1.53)***	1.41 (1.24–1.60)***
Social network size	0.84 (0.76–0.94)**	0.99 (0.84–1.16)
Perceived mattering	0.84 (0.75–0.93)**	0.88 (0.75–1.03)
Dispositional optimism	0.85 (0.76–0.95)**	0.91(0.78–1.08)
SA-PRS	1.21 (1.09–1.35)***	1.16 (1.00–1.35)*
SA-PRS x dispositional optimism	N/A	0.85 (0.74–0.98)*

Values are adjusted odds ratio (95% CI). All independent variables shown were standardized prior to logistic regression, except for age and sex. Models also adjusted for ancestral principal components and (in NSS only) tranche. *p < 0.05, **p < 0.01, ***p < 0.001. *NSS* New Soldier Study, *PPDS* Pre/Post Deployment Study, *SA-PRS* Polygenic risk score for suicide attempt. *N/A* not applicable (effect not estimated because it was non-significant in the preliminary model).

### Multivariable logistic regression model of LSA in the PPDS sample

**Fig. 1:**
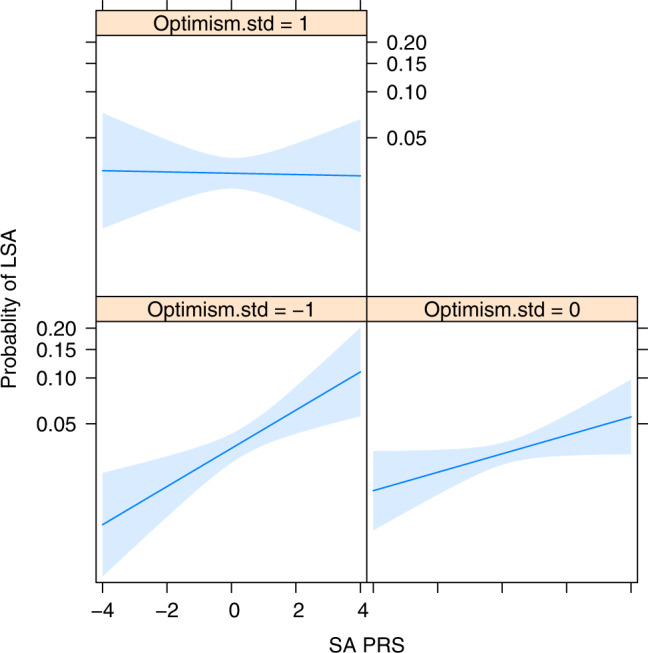
Illustration of the SA-PRS x dispositional optimism interaction effect on odds of lifetime suicide attempt in the Pre/Post deployment study sample (*n* = 4900). The plots show the relationship between SA-PRS and odds of lifetime suicide attempt for those reporting high (1 SD above the mean), average, and low (1 SD below the mean) levels of dispositional optimism.

The multivariable model of LSA within the PPDS sample is shown in Table 4 (panel B). The SA-PRS x dispositional optimism interaction term was significant in the preliminary model, whereas the other interaction effects did not approach statistical significance (AORs = 0.94–1.11, *p*s > 0.19). Thus, the final model estimated the main effects of SA-PRS and environmental and behavioral risk/protective factors, and the SA-PRS x dispositional optimism interaction effect. Results indicated that the association of SA-PRS with LSA in the PPDS sample varied by reports of dispositional optimism [AOR = 0.85 (0.74–0.98), X^2^ = 4.96, *p* = 0.026; see Fig. 1 for a visualization]. For PPDS participants reportin*g* average dispositional optimism, high SA-PRS (1 SD above the sample mean) was associated with a 16% increase in odds of LSA [AOR = 1.16 (1.00–1.35), X^2^ = 3.85, *p* = 0.05]. For those reporting *low* dispositional optimism (1 SD below the sample mean), high SA-PRS was associated with a 37% increase in odds of LSA. Finally, for PPDS respondents reporting high dispositional optimism (1 SD above the sample mean), SA-PRS was not associated with LSA (AOR = 0.99). The results further indicated that higher lifetime trauma burden [AOR = 1.40 (1.22–1.61), X^2^ = 22.93, *p* < 0.001], childhood maltreatment [AOR = 1.25 (1.13–1.40), X^2^ = 16.87, *p* < 0.001], and negative urgency impulsivity [AOR = 1.41 (1.24–1.60), X^2^ = 26.36, *p* < 0.001] were associated with significantly increased odds of LSA. Social network size and perceived mattering were not significantly associated with LSA in the multivariable model.

## Discussion

The SA-PRS calculated for this investigation was significantly associated with lifetime suicide attempt in two independent cohorts of US Army soldiers. The effect sizes were modest, but not substantially weaker than those of environmental and behavioral risk/protective factors included in the analysis. Further, the model results suggested that the effects of the SA-PRS and environmental/behavioral factors were generally additive as opposed to interactive. An exception was an interaction observed in the PPDS sample, indicating that the strength of the relationship between SA-PRS and LSA weakened as the reported level of dispositional optimism increased. Again, however, the size of this interaction effect was small, and it was only observed in one of the two samples.

This study contributes to an emerging literature that aims to establish whether PRS may help identify individuals at elevated risk of suicidal behavior [16, 18, 19, 23, 34, 35]. Based on the effect sizes observed here, we do not anticipate that the SA-PRS considered in isolation would have strong predictive/clinical value. However, our findings lend support to the idea that combining SA-PRS with information pertaining to environmental and behavioral factors may enable more precise suicide risk stratification. To illustrate, we use the model from the larger analytic sample (NSS) and consider SA-PRS in conjunction with other risk factors. The NSS model suggests that a soldier with high SA-PRS (1 SD above the sample mean) and average levels of lifetime trauma, childhood maltreatment, and negative urgency impulsivity has approximately 21% increased risk of LSA relative to a soldier with an average genetic, environmental, and behavioral risk profile. However, in the presence of environmental risk factors (1 SD above average levels of lifetime trauma and childhood maltreatment), a soldier with high SA-PRS is predicted to have more than twice the risk of LSA relative to a soldier with average genetic, environmental, and behavioral risk (AOR = 1.21*1.25*1.36 = 2.1). And when a behavioral risk factor is added in the form of high negative urgency impulsivity (1 SD above the mean), a soldier with high SA-PRS is estimated to have nearly three times the odds of LSA compared to a soldier with average genetic, environmental, and behavioral risk (AOR = 1.21*1.25*1.36*1.38 = 2.8). A caveat is that the available data did not allow us to verify that the variables we conceptualized as environmental and behavioral risk factors preceded the suicide attempt [i.e., it is possible that some events included in the trauma burden score occurred after the suicide attempt(s); or that characteristics such as negative urgency impulsivity differed prior to the onset of suicidal behavior].

Our findings partly converge with results of a previously mentioned study of US military veterans, which found that the highest probability of LSA was observed in veterans with elevated suicidality PRS (i.e., genetic liability for suicidal thoughts or behavior) *and* high lifetime trauma burden [18]. Whereas we observed strictly additive effects of SA-PRS and trauma burden, that prior investigation found a suicidality PRS x trauma burden interaction, providing evidence that trauma may potentiate the effects of genetic vulnerability to suicidality. A variety of methodological differences could explain the partial discrepancy, including the use of different PRS, divergence of sample characteristics (e.g., mean age of the veteran sample was >60 years), and inclusion of different sets of predictors in multivariable models. We also acknowledge the possibility that our study may have been under-powered to detect interactive effects of risk factors on a rare outcome such as suicide attempt.

More robust social networks, perceived mattering, and dispositional optimism were associated with reduced odds of LSA in the NSS model; however, these effects were small and not cross-validated in the PPDS sample. Similarly, the interaction effect involving dispositional optimism in the PPDS sample was not replicated in the NSS sample. These results do not imply that protective factors are inconsequential; however, in models that included multiple risk factors for suicidal behavior, the study measures of social network size, perceived mattering, and dispositional optimism were not consistently associated with LSA. Despite this, it is worth noting a similarity between the SA-PRS x dispositional optimism effect observed in the PPDS sample and a previous finding indicating that dispositional optimism moderated the effect of a suicidality PRS on risk of suicidal ideation among US military veterans [19]. In that study, the association between the suicidality PRS and chronic suicidal ideation (defined as reporting suicidal ideation at both baseline and follow-up) was strongest among veterans who reported low dispositional optimism and weakened as level of optimism increased—with the effect of the suicidality PRS effectively neutralized in those with high optimism [19]. A moderating effect of dispositional optimism on the association between the suicidality PRS and remission of suicidal ideation was also observed. Collectively, results to date may suggest a role for interventions that promote adaptive cognitive styles in mitigating adverse impacts of high genetic risk for suicidal thoughts or behaviors. The findings also imply that high genetic liability for suicidal thoughts or behavior may be of greater concern in the presence of low dispositional optimism, a possibility that merits continued study.

Several study limitations must be noted. First, due to limited availability of reference GWAS data for other populations, we were only able to examine the associations of SA-PRS with risk of LSA among soldiers of European ancestry. Moreover, the analysis samples were primarily comprised of males aged 18–30. Future studies should evaluate if SA-PRS is associated with suicide attempt among individuals of other ancestral backgrounds and in samples with more gender and age diversity. Second, it is likely that a small proportion of participants classified as having no history of LSA had made suicide attempts that were not captured in the administrative or survey data (i.e., prevalence may have been underestimated). A factor to consider in this regard is that some soldiers might have been hesitant to report or seek medical care for suicide attempts due to stigma or career concerns. Third, evaluation of environmental and behavioral factors was based on self-report, a modality that is vulnerable to recall and response biases. Fourth, as noted above, we were unable to establish that the reported personality characteristics, social networks, and trauma exposures predated the suicidal behavior. Fifth, we cannot make assumptions about the mechanisms that explain the associations of SA-PRS and environmental/behavioral factors with suicide attempt (including the extent to which these associations are mediated by mental disorders, a topic that was not addressed in this study). Finally, personality traits were measured with brief scales. Inclusion of more items assessing each domain would likely improve the reliability of the measures and increase power to detect the effects of these characteristics.

In conclusion, we calculated an SA-PRS that was significantly associated with lifetime history of suicide attempt in two cohorts of US Army soldiers. The associations of SA-PRS with increased odds of LSA remained significant in models that also included environmental and behavioral risk/protective factors. Furthermore, the effects of SA-PRS and the environmental/ behavioral factors were largely additive, as opposed to interactive. Overall, the study findings suggest that SA-PRS may contribute unique information for the purpose of suicide risk stratification. However, given the small effect magnitudes, the cost and incremental benefits of utilizing SA-PRS for risk targeting must be given careful consideration.
